# PTBP1 and PTBP2 impaired autoregulation of SRSF3 in cancer cells

**DOI:** 10.1038/srep14548

**Published:** 2015-09-29

**Authors:** Jihua Guo, Jun Jia, Rong Jia

**Affiliations:** 1 Hubei-MOST KLOS & KLOBME, School & Hospital of Stomatology, Wuhan University, Wuhan, 430079, PR China

## Abstract

Splicing factors are key players in the regulation of alternative splicing of pre-mRNAs. Overexpression of splicing factors, including SRSF3, has been strongly linked with oncogenesis. However, the mechanisms behind their overexpression remain largely unclear. Autoregulation is a common mechanism to maintain relative stable expression levels of splicing factors in cells. SRSF3 regulates its own expression by enhancing the inclusion of an alternative exon 4 with an in-frame stop codon. We found that the inclusion of SRSF3 exon 4 is impaired in oral squamous cell carcinoma (OSCC) cells. PTBP1 and PTBP2 bind to an exonic splicing suppressor in exon 4 and inhibit its inclusion, which results in overexpression of full length functional SRSF3. Overexpression of SRSF3, in turn, promotes PTBP2 expression. Our results suggest a novel mechanism for the overexpression of oncogenic splicing factor *via* impairing autoregulation in cancer cells.

Alternative splicing of pre-mRNA dramatically increases proteomic diversity and must be precisely regulated. Misregulation of alternative splicing has been identified as the cause of multiple cancers^1^. Splicing factors play key roles in the regulation of alternative splicing. A number of studies have reported that some splicing factors can function as oncogenes, including SRSF1^2^, SRSF6^3^,^4^, and SRSF3^5^. SRSF3, also called SRp20 or SFRS3, is the smallest member of the serine/arginine (SR)-rich protein family^6^. SRSF3 has multiple cellular functions, including alternative splicing^7^, termination of transcription^8^, alternative RNA polyadenylation^9^, protein translation^10^, and RNA export^11^,^12^. SRSF3 has also been reported to be associated with chromatin^13^, and is essential for the differentiation and metabolic function of hepatocytes^14^. SRSF3 activates the inclusion of exons in many alternative splicing events. It has also been demonstrated that SRSF3 plays a negative role in exon inclusion^15^,^16^.

SRSF3 has been found to be involved in a number of human diseases^17^. Previously we demonstrated that SRSF3 is a proto-oncogene^5^ and frequently overexpressed in multiple cancers^18^,^19^. However, as in the case of other oncogenic splicing factors, the causes of its overexpression remain largely unclear. In this study, we used oral squamous cell carcinoma (OSCC) as a model to investigate the potential causes of SRSF3 overexpression. It has been reported that SRSF3 regulates its own expression by enhancing the inclusion of exon 4 in mouse cells. Since exon 4 has an in-frame pre-mature stop codon, inclusion of this exon suppresses the expression of full length SRSF3. The alternative exon 4 of human SRSF3 has also been annotated in databases, including RefSeq (accession number: NR_036610.1), UCSC Genes (uc003omk.3), and ENSEMBL (accession number: ENST00000477442). The regulation mechanisms of alternative exon 4 of human SRSF3 remain unknown. We found that PTBP1 and PTBP2 impair SRSF3 autoregulation and enhance SRSF3 expression by inhibiting the inclusion of exon 4 *via* interactions with an exonic splicing suppressor.

## Results

### Expression and function of SRSF3 in oral squamous cell carcinoma

**Figure 1:**
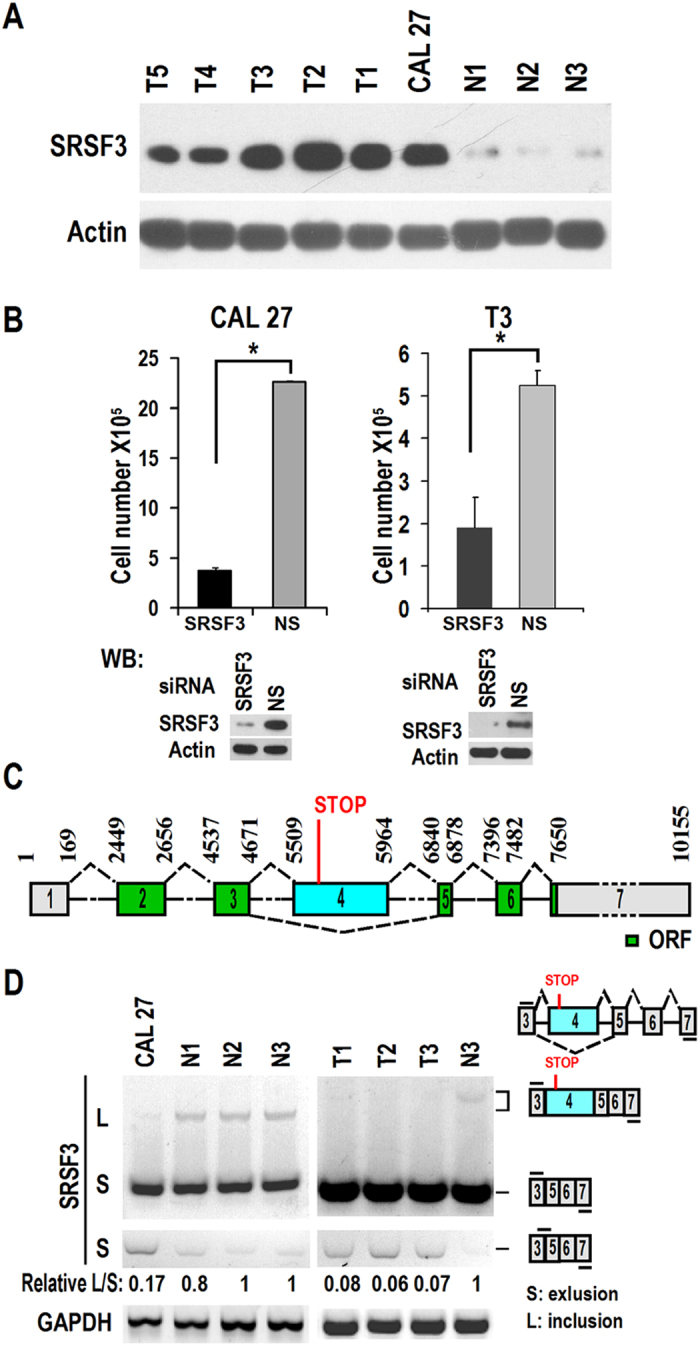
Autoregulation of SRSF3 expression is disturbed in OSCC cancer cells. (**A**) SRSF3 is overexpressed in oral squamous cell carcinoma cell lines. Western blot analysis showed the expression of SRSF3 in primary human oral squamous carcinoma cells (T1 to T5), CAL 27 or normal primary oral mucosal epithelial cells (N1 to N3). β-actin served as loading control. (**B**) SRSF3 is required for the growth of oral squamous cell carcinoma cells. A total of 5 × 10^5^ CAL 27 or T3 cells were inoculated into 6 well plates and transfected by 20 nM SRSF3 siRNA (#1) or non-specific siRNA with Lipofectamine 2000 on Day 0. Cells were passed and received another transfection on Day 2. Cell numbers were counted on Day 4. Western blot analysis showed knockdown efficiency of SRSF3. *p < 0.05. (**C**) Schematic diagram of the pre-mRNA and splicing pattern of human SRSF3. Human SRSF3 pre-mRNA contains an alternatively spliced exon 4 between exon 3 and exon 5. There is an in-frame stop codon in the 5′ end of exon 4. The numbers above the pre-mRNA exons (boxes) and introns (lines) are the nucleotide positions in pre-mRNA. Dashed lines above or below introns indicate RNA splicing direction. (**D**) Alternative splicing of exon 4 in OSCC cells (CAL 27, and T1-T3) or normal primary oral mucosal epithelial cells (N1 to N3) was analyzed by RT-PCR with primers located in the constant exon 3 and 7. Because SRSF3 transcript without exon 4 (S) is much more than that with exon 4 (L) in cells, an exon 3/5 forward junction primer was also used to specifically amplify exon 4-excluded short product. Relative L/S represents the ratio of band intensities of long *vs* short isoforms. GAPDH served as loading control. Diagrams on the right of (**D**) show the structures of human SRSF3 pre-mRNA and spliced products and primer positions (short lines above or below exons).

Previously we found that SRSF3 is a proto-oncogene that is overexpressed in multiple cancers^5^. However, the expression and function of SRSF3 in oral carcinoma is unknown to date. We isolated and purified 5 primary OSCC cells and 3 normal primary oral mucosal epithelial cells. Western blot analysis showed that an OSCC cell line CAL 27 and primary OSCC cells have significant upregulation of SRSF3 compared to normal cells (Fig. 1A). We also verified SRSF3 overexpression in a tissue array (including 50 OSCC tumor and 10 normal oral mucosal samples) by immunohistochemistry (Figure S2). Knockdown of SRSF3 in CAL 27 and a primary OSCC cell T3 with SRSF3 siRNA significantly inhibited cell growth compared with controls transfected with non-specific siRNA (Fig. 1B). These results indicated that SRSF3 is also overexpressed in OSCC cells and required for their growth.

### Alternative splicing of exon 4 in the human SRSF3 gene

It has been reported that mouse SRSF3 gene contains an alternative exon 4^20^,^21^. As in mice, human SRSF3 gene also has an alternative exon 4 (Fig. 1C). Transcripts without the alternative exon 4 encode full length SRSF3. However, since exon 4 contains a stop codon, inclusion of this exon may result in truncation of the SRSF3 protein lacking of arginine/serine-rich domain (Figure S1). We analyzed the alternative splicing of the exon 4 in CAL 27 and normal primary oral mucosal epithelial cells. RT-PCR showed obvious inclusion of the exon 4 in normal cells. However, the inclusion of this exon significantly reduced in CAL 27 and primary OSCC cancer cells (Fig. 1D). These results indicated that inclusion of exon 4 was impaired in OSCC cancer cells, which might result in overexpression of full length functional SRSF3.

### Exonic splicing suppressor in exon 4 of human SRSF3 gene

**Figure 2:**
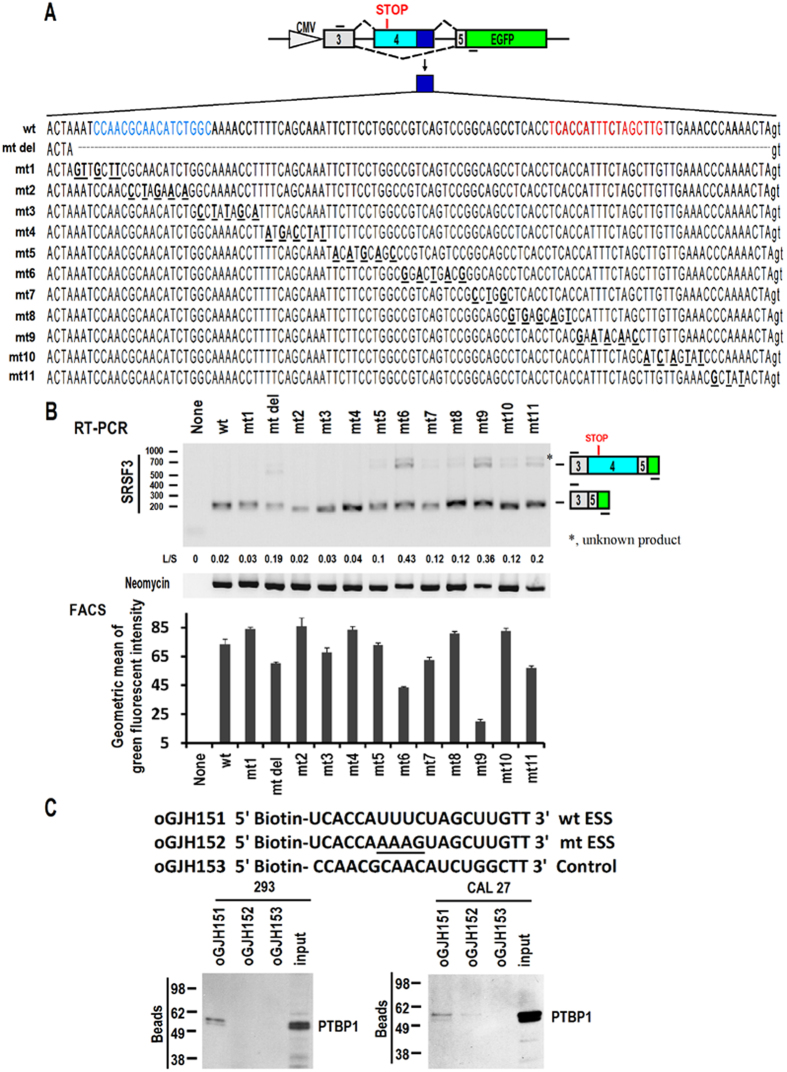
PTBP1 interacts with the ESS motif of SRSF3 exon 4. (**A**) Diagram of SRSF3 minigene. Genomic sequence from 3′ part of exon 3 to 5′ part of exon 5 of SRSF3 was amplified from CAL 27 ells and cloned into pEGFP-N1. A kozak consensus sequence and an in-frame ATG were added to the 5′ end of minigene. GFP is allowed to be translated in the absence of exon 4. To mapping potential regulatory motifs, the 3′ part of alternative exon 4 was deleted or serially mutated. (**B**) Wild type (wt) or mutated (mt) minigenes were transfected into 293 cells. Alternative splicing of exon 4 were analyzed by RT-PCR. L/S represents the ratio of band intensities of long *vs* short isoforms. Neomycin resistant gene served as loading and transfection efficiency control. GFP fluorescent intensities in transfected cells were also analyzed by FACS. The diagram below neomycin showed the geometric means of green fluorescent intensities of minigene transfected cells (duplicate samples for each plasmid). (**C**) RNA pulldown assay showed that PTPB1 interacts with the ESS motif of SRSF3 alternative exon 4. Biotinylated oligo RNAs [including wt (oGJH151) or mt (oGJH152) ESS motif based on minigene mt9, and a control sequence (oGJH153) based on (mt1 and mt2)] were incubated with 293 or CAL 27 total cellular extract. Binding proteins were separated in SDS-PAGE gel and blotted with mouse anti-PTBP1 antibody. In panel A, the corresponding positions of oGJH151 and oGJH153 in exon 4 were labeled red and blue, respectively.

Next, we sought to understand the mechanisms by which inclusion of exon 4 was impaired. Previously Jumaa *et al.* showed that deletion of the 3′ end of the mouse SRSF3 exon 4 promoted its own inclusion^20^. Therefore, we speculated that there might be an exonic splicing suppressor (ESS) in this region in the human SRSF3 gene. We constructed a minigene containing genomic sequence from exon 3 to exon 5, including exon 4. An ATG was added to start translation of exon 3, exon 5 and GFP, and express a SRSF3-GFP fusion protein. However, inclusion of exon 4 will introduce a stop codon and quench the expression of GFP. We found that deletion of the 3′ end of human SRSF3 exon 4 (mt del) also promoted its inclusion (Fig. 2A,B). We performed point mutation in this region to map potential ESS sites. Two mutations, mt6 and mt9, showed significant increases in the inclusion of exon 4 (Fig. 2B). FACS analysis of GFP intensity showed that mt9 resulted in the strongest reduction in GFP expression (Fig. 2B). Based on this result, we deduced that mt9 must be located in an ESS motif.

### The ESS motif is associated with PTBP1

Next, we sought to find the splicing factors associated with the ESS. In the middle of the mt9 sequence is “UUUCU”, which is similar to the motifs identified by PTBP1, such as “UCUUC”^22^. RNA pulldown assay showed that PTBP1 binds to the ESS motif in both 293 and CAL 27 cells *in vitro*. Mutation from “UUUCU” to “AAAGU” abolished the association of PTBP1 and the ESS. A control sequence upstream of mt9 showed no interaction with PTBP1 (Fig. 2C).

### PTBP1 and PTBP2 inhibits the inclusion of exon 4

**Figure 3:**
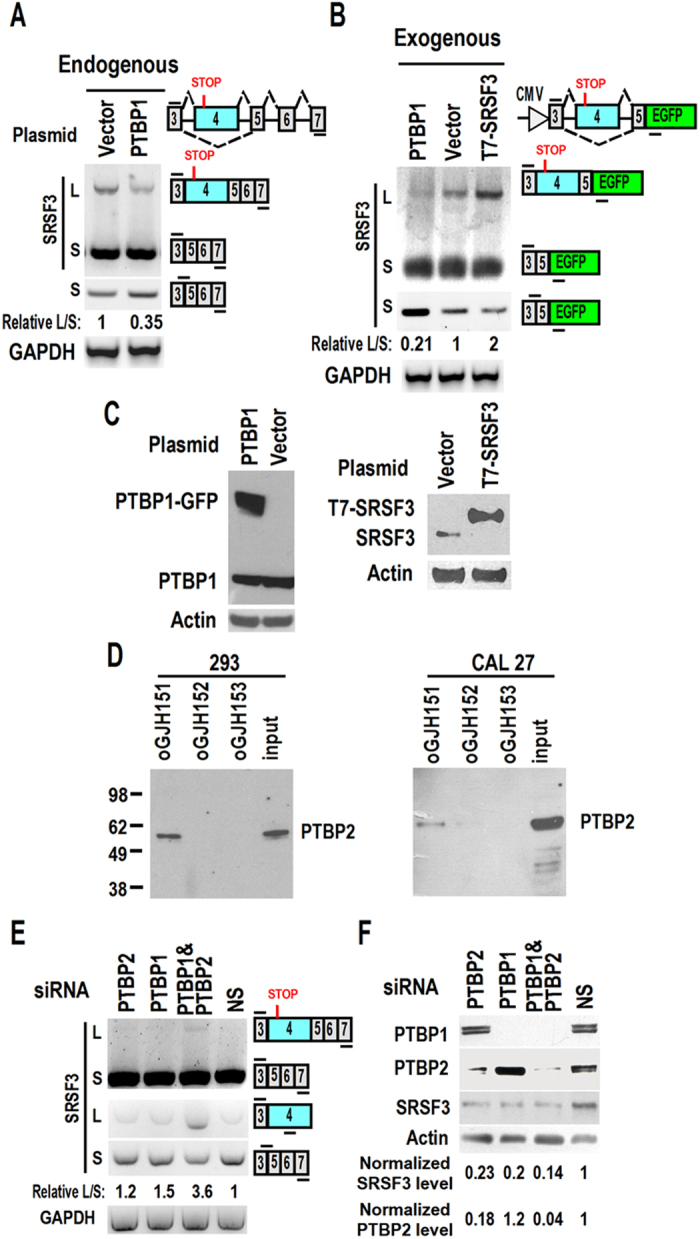
PTBP1 and PTBP2 inhibit the inclusion of SRSF3 exon 4. (**A–B**) Overexpression of PTBP1 inhibited inclusion of exon 4. 293 cells were transfected with PTBP1 expression plasmid (**A**) or co-transfected with minigene and PTBP1 expression plasmid (**B**). Alternative splicing of exon 4 were analyzed by RT-PCR with primers located in the constant exon 3 and 7. An exon 3/5 forward junction primer was used to specifically amplify exon 4 excluded short product. Relative L/S represents the ratio of band intensities of long *vs* short isoforms. GAPDH served as loading control. Diagrams on the right of (**A**,**B**) show the structures of SRSF3 pre-mRNA and spliced products and primer positions (short lines above or below exons). (**C**) Overexpression of PTBP1 (fused with GFP) and T7 tagged SRSF3 were confirmed by western blot with specific anti-PTBP1 or SRSF3 antibody. (**D**) PTBP2 interacts with the ESS motif of SRSF3 exon 4. The western blot membranes in Fig. 2C was stripped and blotted with specific anti-PTBP2 antibody. (**E–F**) Knockdown of PTBP1 and PTBP2 promoted inclusion of exon 4. 293 cells were transfected by 20 nM PTBP1 (#1) and/or PTBP2, or non-specific (NS) siRNA. Alternative splicing of exon 4 were analyzed by RT-PCR with primers located in the constant exon 3 and 6 (**E**). Exon 4 specific and exon 3/5 forward junction primer were also used to specifically detect exon 4-included (L) and excluded (S) products, respectively (**E**). Western blot analysis showed the knockdown efficiency of PTBP1 and PTBP2 (**F**). Expression of SRSF3 is affected by knockdown of PTBP1 and PTBP2.

We then investigated the function of PTBP1 in alternative splicing of exon 4 *in vivo*. We transfected 293 cells with PTBP1 expression plasmid. Overexpression of PTBP1 significantly reduced the inclusion of exon 4 (Fig. 3A,C). Co-transfection of the minigene and PTBP1 expression plasmid in 293 cells also showed the similar results (Fig. 3B). In addition, as expected, overexpression of SRSF3 autoregulated its own expression by increasing inclusion of exon 4, similar to the result previously found in the mouse SRSF3 gene (Fig. 3B,C)^20^.

However, knockdown of PTBP1 with siRNA had no effect on the alternative splicing of exon 4 (Figure S3). We speculated that other splicing factors might also inhibit the inclusion of exon 4. PTBP2 is a homolog of PTBP1 and they have similar functions^23^. PTBP2 has been shown to compensate for the loss of PTBP1^24^. Western blot assay showed that ESS motif also interacts with PTBP2 in both 293 and CAL 27 cells *in vitro* (Fig. 3D). Knockdown of both PTBP1 and PTBP2 increased the inclusion of exon 4 (Fig. 3E,F). We also found by western blot that knockdown of both PTBP2 and PTBP1 reduced the expression level of SRSF3. Surprisingly, knockdown of either PTBP2 or PTBP1 alone also reduced the protein level of SRSF3, suggesting that these two splicing factors may also be involved in other mechanisms of regulating SRSF3 expression. As expected, knockdown of both PTBP1 and PTBP2 yielded the lowest level of SRSF3 (Fig. 3F). Collectively, these results indicated that PTBP1 and PTBP2 interact with an ESS motif and inhibit the inclusion of exon 4 in SRSF3 transcripts.

In addition, previous studies showed that PTBP1 negatively regulates the expression of PTBP2^24^,^25^. We also found that depletion of PTBP1 slightly increased the expression of PTBP2 (PTBP1 siRNA *vs* NS siRNA, Fig. 3F).

### Expression of SRSF3 correlates with PTBP1 and PTBP2 in cancer cells

**Figure 4:**
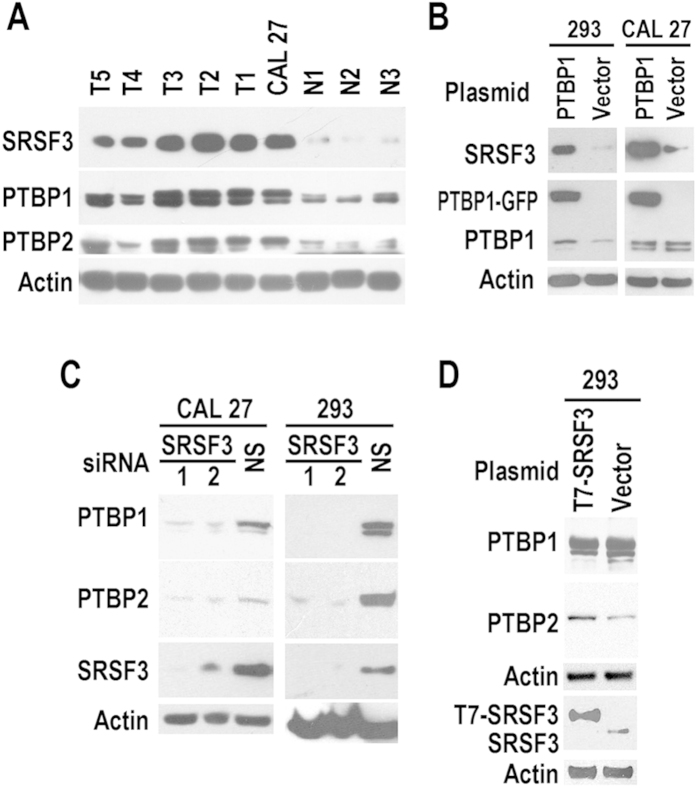
Co-expression and mutually regulation of SRSF3, PTBP1, and PTBP2 in OSCC cells. (**A**) Expression of SRSF3, PTBP1, and PTBP2 in tumor and normal cells. Western blot membrane in Fig. 1A was further probed by specific anti-PTBP1 or PTBP2 antibody. T1 to T5 are primary oral squamous carcinoma cells. N1 to N3 are primary normal oral mucosal epithelial cell. (**B**) Overexpression of PTBP1 promoted SRSF3 expression in 293 or CAL 27 cells. Cells were transfected by PTBP1 (fused with GFP) expression plasmid or empty vector. (**C**) Knockdown of SRSF3 repressed the expression of PTBP1 and PTBP2 in 293 or CAL 27 cells. Cells were transfected by 20 nM SRSF3 siRNA or non-specific (NS) siRNA. The expression of PTBP1 and PTBP2 were analyzed by western blot. (**D**) Overexpression of SRSF3 promoted the expression of PTBP2. Western blot was used to analyze the expression of PTBP1 and PTBP2 in 293 cells transfected by T7 tagged SRSF3 expression plasmid or empty vector in Fig. 3C. β-actin served as loading control.

Next, we analyzed the relationship between SRSF3 and its regulators in OSCC cells. Primary OSCC cells and CAL 27 cancer cell line had higher level of PTBP1 and PTBP2 than normal cells (Fig. 4A). Overexpression of PTBP1 increased SRSF3 expression in both 293 and CAL 27 cells (Fig. 4B). These results confirm the association of SRSF3 expression with PTBP1 and PTBP2.

### SRSF3 regulates the expression of PTBP1 and PTBP2

Mutually regulation is common in splicing factors. We then investigated whether SRSF3 regulates the expression of PTBP1 and PTBP2. Knockdown of SRSF3 repressed the expression of PTBP1 and PTBP2 in 293 or CAL 27 cells (Fig. 4C). Overexpression of SRSF3 promoted the expression of PTBP2, not PTBP1 (Fig. 4D). These results indicated that the expression of PTBP1 and PTBP2 also requires SRSF3. SRSF3 may positively feed back to the expression of PTBP2.

### Exon 4-included transcripts are nonsense-mediated mRNA decay (NMD) targets

**Figure 5:**
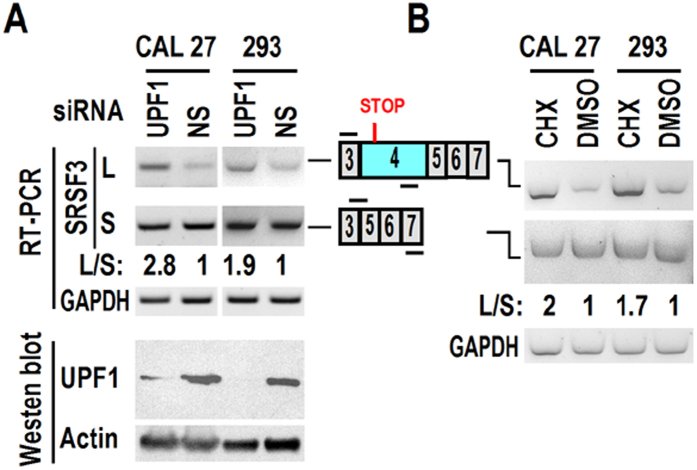
SRSF3 transcripts with exon 4 are NMD targets. (**A**) CAL 27 or 293 cells were transfected with 20 nM UPF1 or non-specific (NS) siRNA twice in a 48-hour interval. Alternative splicing of SRSF3 exon 4 was analyzed by RT-PCR. Exon 4 specific and exon 3/5 forward junction primers were used to detect transcripts with exon 4 inclusion (L) and exclusion (S), respectively. Western blot analysis showed knockdown efficiency of UPF1. (**B**) NMD was inhibited indirectly by treating CAL 27 or 293 cells with 100 μg/ml CHX (Cell Signaling Technology, USA) for 5 h. Alternative splicing of SRSF3 exon 4 was analyzed by RT-PCR.

Expression level of exon 4-included transcript is much less than exon 4-excluded transcript (Figs 1D and 3A,B). Since inclusion of exon 4 introduces a premature stop codon, we speculated that exon 4-included transcript might be degraded *via* NMD. We used cycloheximide (CHX) and UPF1 siRNA for indirect and direct NMD inhibition, respectively. Exon 4-included transcript significantly increased upon inhibition of NMD (Fig. 5A,B). Therefore, this transcript is the target of NMD. However, we cannot exclude this possibility that remaining undegraded transcripts are able to encode truncated SRSF3. Currently, we cannot detect truncated SRSF3 by western blot with commercial available antibody.

**Figure 6:**
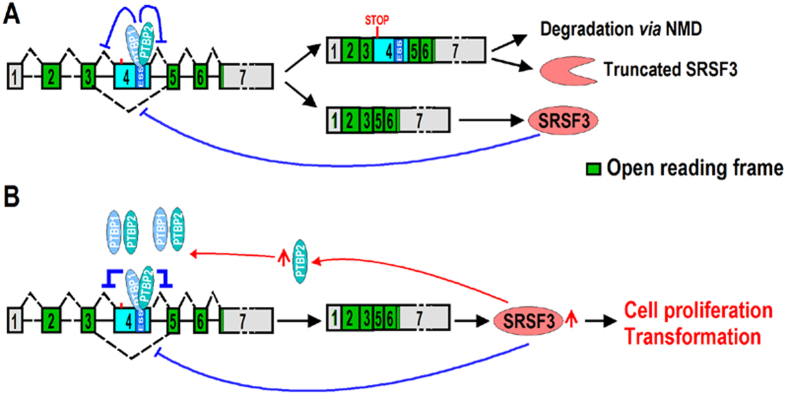
Model of SRSF3 expression regulated by PTBP1 and PTBP2 *via* controlling the alternative splicing of exon 4. (**A**) The exon 4-excluded transcripts encode full length functional SRSF3. The exon 4-included transcripts were degraded *via* the NMD pathway, or may produce truncated SRSF3. In normal cells, SRSF3 regulates its own expression by inhibiting the exclusion of exon 4. (**B**) In cancer cells, overexpressed PTBP1 and PTBP2 bind to ESS motif and impair inclusion of exon 4, which then increases the expression of full length SRSF3 and promotes cell proliferation and transformation. Meantime, SRSF3, in turn, also promotes the expression of PTBP2.

Taking all of our data together, we propose a new model of regulation of SRSF3 expression (Fig. 6). Expression of SRSF3 is regulated by PTBP1 and PTBP2 *via* alternative splicing of exon 4 of SRSF3. Cancer cells take advantage of high levels of PTBP1 and PTBP2 to impair the inclusion of exon 4, thereby allowing the overexpression of full length SRSF3 and promoting cell proliferation and transformation. PTBP2 may also be involved in this autoregulatory loop *via* a positive feedback mechanism.

## Discussion

A growing body of evidence has demonstrated that some splicing factors function as oncogenes, such as SRSF1^2^, SRSF6^3^,^4^, and SRSF3^5^. Overexpression of these splicing factors can promote cell growth and transform cells. However, the mechanisms behind the overexpression of these splicing factors remain largely unknown. Gene amplification could be such mechanism.

In a previous study, we concluded that SRSF3 is a proto-oncogene^5^. However, it is still unclear how SRSF3 is overexpressed in cancerous cells. We searched the oncomine database (www.oncomine.org) for SRSF3 DNA amplification detection in cancers. However, no evidence of significant more than 1.5 fold increase in SRSF3 gene DNA copy number compared with normal controls was found. Therefore we concluded that DNA amplification might not be the main cause of SRSF3 overexpression.

Most human genes undergo alternative splicing of pre-mRNA to produce two to thousands of transcripts. Different transcripts of a gene may function differently or conversely, and so alternative splicing of genes need to be regulated precisely to ensure generation of proper transcripts for normal physiological activity of cells. Both decreased and increased expression of SRSF3 will significantly change the normal profile of alternative splicing in cells. Hence, in normal cells, expression of SRSF3 must be strictly regulated. SRSF3 regulates alternative splicing of the exon 4 in its own mRNA. The long isoform with exon 4 contains an in-frame pre-mature stop codon, will be degraded *via* NMD or may encode a non-functional truncated SRSF3 protein lacking RS domain^20^. Upregulation of SRSF3 promotes the inclusion of exon 4, which down-regulates the availability of full length SRSF3. Cells use this autoregulation mechanism to maintain a stable level of SRSF3. In the present study, we discovered that cancer cells exhibit decreased inclusion of exon 4 and increased amount of SRSF3, suggesting that autoregulation of SRSF3 is impaired in those cells (Fig. 1D).

Autoregulation seems to be common among serine/arginine-rich (SR) splicing factors. SRSF1 has a similar regulatory mechanism as that of SRSF3. Alternative splicing of intron 3 and exon 4 of SRSF1 produces 6 isoforms. Overexpression of SRSF1 promotes inclusion of intron 3 or partial deletion of exon 4, which then decreases the amount of the isoform encoding full length functional SRSF1^26^. Overexpression of SRSF2 induces both one exon inclusion and one intron excision in the 3′UTR region of SRSF2. The resulting transcript is unstable, which then causes decrease in the SRSF2 protein level^27^. In the present study, we demonstrate for the first time that cancer cells have impairment in this autoregulation mechanism and obtain higher level of oncogenic splicing factors compared to normal cells.

Alternative splicing of pre-mRNA is mainly regulated by splicing factors. Jumaa *et al.* found that SRSF1 plays a negative role in the inclusion of exon 4 in the mouse SRSF3 gene^20^. Later, they also demonstrated that ASF3 (an SRSF1 isoform without the RS region) plays a similar role as SRSF1, and that SFRS10, SRSF7 and SRSF5 have no effect on alternative splicing of SRSF3 exon 4^21^. However, the exact sequences interacting with these splicing factors remained unknown. In the present study, we found that two other splicing factors, namely PTBP1 and PTBP2, also inhibit the inclusion of SRSF3 exon 4. These two factors belong to heterogeneous nuclear ribonucleprotein (hnRNPs) family. It has been reported that PTBP1 binds to a pyrimidine-rich ESS motif in bovine papillomavirus type 1^28^. We found that an ESS motif in SRSF3 exon 4 interacts with these two factors. The sequence of this ESS motif is pyrimidine-rich and similar to the motifs known to interact with PTBP1.

SRSF3 plays essential roles in multiple cellular processes. Loss of SRSF3 is embroynic lethal^29^. Even though overexpression of SRSF3 have been reported in multiple cancers, a recent research reported that a mouse line with hepatocyte-specific deletion of SRSF3 developed spontaneous hepatocellular carcinoma in adulthood^30^. This suggests that SRSF3 has essential roles not only in development and oncogenesis but also in anti-oncogenesis in some cell types. Therefore, maintaining normal levels of SRSF3 or other splicing factors in a specific cell type is critical for preventing cell transformation.

In summary, we found that autoregulaton of SRSF3 expression involves inclusion of exon 4, which is regulated by PTBP1 and PTBP2 binding to an ESS motif, and that this regulatory process is impaired in OSCC cancer cells.

## Methods

### Tissues and Cells

OSCC tissues were collected from the School of Stomatology in Wuhan University. Primary OSCC cells (T1, T2, T3, T4, and T5) were cultured in Dulbecco’s modified Eagle medium (DMEM; HyClone, USA) supplemented with 10% fetal bovine serum (FBS, Hyclone, USA) and 1% antibiotic-antimycotic (Gibco, USA). Cancer cells were purified by removing fibroblasts with a short treatment of 0.25% trypsin-EDTA (Invitrogen, USA). Normal gingival epithelial cells (N1, N2, and N3) were collected from gingival tissues of healthy donors and grown in keratinocyte growth medium (KGM, Lonza, Switzerland). To compare the expression of SRSF3, all cancer cells were treated with KGM for two days. Informed consent was obtained from all subjects, and all experimental protocols were approved by the Ethics Committee at the School of Stomatology in Wuhan University. The methods were carried out in accordance with the approved guidelines. CAL 27 is an OSCC cell line. HEK-293 is a human embryonic kidney epithelial cell line. CAL 27 and HEK-293 cells were grown in DMEM medium with 10% FBS and 1% antibiotic-antimycotic drugs.

### Plasmids

Genomic DNA sequence from exon 3 to exon 5 was amplified from the CAL 27 genome with primer oGJH45 5′ GCCGTGTAAGAGTGGAACTGTCG 3′ and oGJH46 5′ AAACACTGCCATTTCTAAGAACGTAG 3′. Then, the start codon (ATG), and the restriction sites EcoRI and BamHI were added to exon 3 and exon 5 using primers oGJH65 5′ GCT**GAATTC**ACC**ATG**GGCCGTGTAAGAGTGGAACTG 3′ and oGJH66 5′ GGT**GGATCC**CGGCTGCGAGAGAAGC 3′. The PCR product amplified by oGJH65 and oGJH66 was cloned into pEGFP-N1 at EcoRI and BamHI sites. The resulting plasmid carried genomic minigene of SRSF3 exon 3 to exon 5, including exon 4. Since SRSF3 exon 3 and exon 5 are in the same open reading frame as GFP, this plasmid would result in expression of a SRSF3-GFP fusion protein. However, inclusion of exon 4 will add a premature stop codon and prevent the expression of GFP.

The PTBP1 expression plasmid was bought from Genechem (China). The open reading frame of PTBP1 was fused with GFP. The T7-SRSF3 expression plasmid was kindly provided by Dr. Zheng Zhi-Ming (National Cancer Institute, USA).

### RNAi

Human PTBP1, PTBP2, UPF1 and non-specific siRNA were synthesized by GenePharma (China). The sequences of siRNAs used in the study are as follows: 5′ AGGUGACAGCCGAAGUGCA 3′ (PTBP1 #1), 5′ UGACAAGAGCCGUGACUAC 3′ (PTBP1 #2)^31^, 5′ GCUGUUAUCAUUCCUUGGUUA 3′ (PTBP2), 5′ AAGAUGCAGUUCCGCUCCAUU 3′ (UPF1)^32^. SRSF3 #1 and SRSF3 siRNA #2 were bought from Ambion and Santa Cruz Biotechnology, respectively. A total of 5 × 10^5^ CAL 27, T3, or 293 cells were seeded into 6-well plates and transfected with 20 nM siRNA in the presence of Lipofectamine 2000 (Invitrogen, USA) according to the manufacturer’s instructions. After 2 days, cells were passed and received another transfection. After 4 days, cell numbers were counted. Total protein and RNA were also collected.

### Western blot

Total protein samples were boiled and separated in 10% SDS-PAGE gel and transferred to a nitrocellulose membrane. The membrane was blotted with the following antibodies: monoclonal mouse anti-SRSF3 (clone 7B4, invitrogen, USA), monoclonal mouse anti-PTBP1 (Santa Cruz Biotechnology, USA), monoclonal rabbit anti-PTBP2 (Epitomics, USA), monoclonal rabbit anti-UPF1 (Cell Signaling Technology, USA), or horseradish peroxidase-labeled mouse anti-β-actin antibody (Sigma-Aldrich).

### RNA preparation and reverse transcriptase PCR (RT-PCR)

Total RNA was extracted from cells using the Total RNA Miniprep Kit (AxyPrep, USA). One microgram total RNA was treated with DNase I (Invitrogen) to eliminate DNA contamination, and then reverse transcribed using random hexamers with the Superscript II reverse transcriptase (Invitrogen, USA) at 37 °C. PCR was performed with the following primer pairs: oGJH 209 5′ GGAGTCCTCCACCTCGTCGCA 3′ and oGJH 212 5′ ACGAGACCTAGAGAAGGATCGGGAC 3′ for endogenous exon 4 alternative splicing detection; oGJH209 and oGJH101 5′ GCTCCTCGCCCTTGCTCACCA 3′ for exogenous exon 4 alternative splicing detection in the minigene; oGJH761 5′ AGTCCTCCACCTCGTCGCAGATCTC 3′ (exon 3 and 5 junction primer) and oGJH213 5′ CCATAGAGAATTACACCTTTGTGTCACTG 3′ (exon 7) for exon 4-skipped SRSF3; oGJH211 5′ CTCCCTCTTGGGGTCGTCGC 3′ and oGJH759 5′ CATGTGAAACGACACCAGCCAAGC 3′ for exon 4-included SRSF3; oGJH102 5′ ATTGAACAAGATGGATTGCACGC 3′ and oGJH103 5′ TCAAGAAGGCGATAGAAGGCG 3′ for neomycin resistant gene; 5′-GTCATCAATGGAAATCCCATCACC-3′ and 5′-TGAGTCCTTCCACGATACCAAA-3′ for GAPDH.

### GFP fluorescence assay

The fluorescence intensity of GFP in transfected cells was analyzed with a FACS- caliber instrument (BD Biosciences, USA).

### RNA pull-down assay

Biotin-labeled SRSF3 RNA oligonucleotides oGJH151 (5′ Biotin-UCACCAUUUCUAGCUUGTT 3′ wild type exon 4 ESS), oGJH152 (5′ Biotin-UCACCAAAAGUAGCUUGTT 3′, mutant exon 4 ESS, mutation was underlined), and oGJH153 (5′ Biotin- CCAACGCAACAUCUGGCTT 3′, control sequence in exon 4) were synthesized by Takara (Dalian, China). The total CAL 27 cell extract was prepared using radioimmunoprecipitation assay (RIPA) buffer (Sangon Biotech, China). One microliter biotin-labeled RNA oligonucleotide (20 μM) was immobilized onto 10 μl of NeutrAvidin beads (Pierce, USA). The beads were then incubated with 100 μl of CAL 27 total cell extract in 1× binding buffer (20 mM Tris, 200 mM NaCl, 6 mM EDTA, 5 mM potassium fluoride, 5 mM β-glycerophosphate, 2 μg/ml aprotinin, pH 7.5) in a final volume of 400 μl at 4 °C for 2 h. The beads were washed three times with 1×binding buffer. The bound proteins were eluted by sodium dodecyl sulfate sample buffer and analyzed by western blot.

### Tissue Microarray and immunohistochemistry

A human OSCC tissue microarray, containing 50 OSCC and 10 normal oral mucosal tissues, was bought from Alenabio Co., Ltd. (China). Immunohistochemistry (IHC) was performed using the Vector immunodetection kit (Vector Laboratories, USA). Sections were incubated with monoclonal mouse anti-SRSF3 (clone 7B4, invitrogen) overnight at 4 °C. SRSF3 specific nuclear expression in cancer or normal mucosal epithelia was scored into 4 levels (0 = less than 10% positive cells, 1 = 10%–33% positive cells, 2 = 34%–66% positive cells, 3 = more than 66% positive cells). Two independent observers evaluated IHC results. Disagreements were resolved by re-evaluation and discussion.

### Nonsense-mediated mRNA decay inhibition

We used UPF1 siRNA to diretly inhibit NMD. Cells were treated with 20 nM siRNA twice in a 48-hour interval. After 96 hours, total protein and RNA were collected. NMD was also inhibited indirectly by treating CAL 27 or 293 cells with 100 μg/ml cycloheximide (CHX, Cell Signaling Technology, USA) for 5 h.

### Statistical analysis

For cell growth, two-group statistical comparisons of means were calculated with student’s *t*-test using Excel (Microsoft). The scores of SRSF3 in tissue array were compared between groups using the nonparametric Mann-Whitney *U*-test in SPSS software.

## Additional Information

**How to cite this article**: Guo, J. *et al.* PTBP1 and PTBP2 impaired autoregulation of SRSF3 in cancer cells. *Sci. Rep.* **5**, 14548; doi: 10.1038/srep14548 (2015).

## Supplementary Material

Supplementary Figures
