# Supplementary material for: PTBP1 and PTBP2 impaired autoregulation of SRSF3 in cancer cells
Source: Sci Rep. 2015 Sep 29;5:14548. doi: 10.1038/srep14548 (PMC4586487; doi:10.1038/srep14548)

Title: PTBP1 and PTBP2 impaired autoregulation of SRSF3 in cancer cells
Authors: Jihua Guo, Jun Jia, Rong Jia

Supplementary Figure legends

Figure S1

Open reading frames of SRSF3 with or without exon 4.

Figure S2

SRSF3 is overexpressed in OSCCs. The expression of SRSF3 was analyzed in a tissue array (50 OSCC tumor and 10 normal oral mucosal samples) by immunohistochemistry. (**A**) Representative immunohistochemical staining results of SRSF3 in OSCC or normal oral mucosal samples. (**B**) Immunostaining scores of SRSF3 in normal or tumor tissues of the tissue array.

Figure S3

Knockdown of PTBP1 had no effect on the alternative splicing of exon 4. (**A**) CAL 27 cells were transfected with 20 nM PTBP1 or non-specific (NS) siRNA twice in a 48-hour interval. Alternative splicing of SRSF3 exon 4 was analyzed by RT-PCR. (**B**) Western blot showed knockdown efficiency of PTBP1.

Figure S1

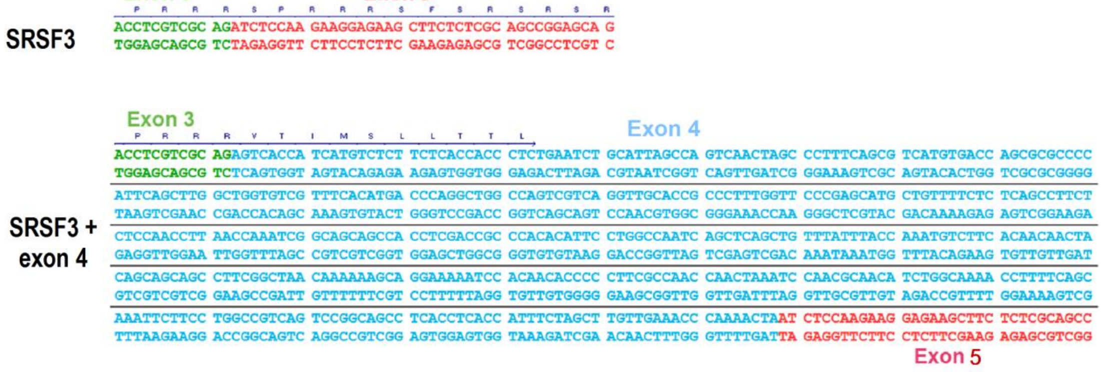

Figure S2

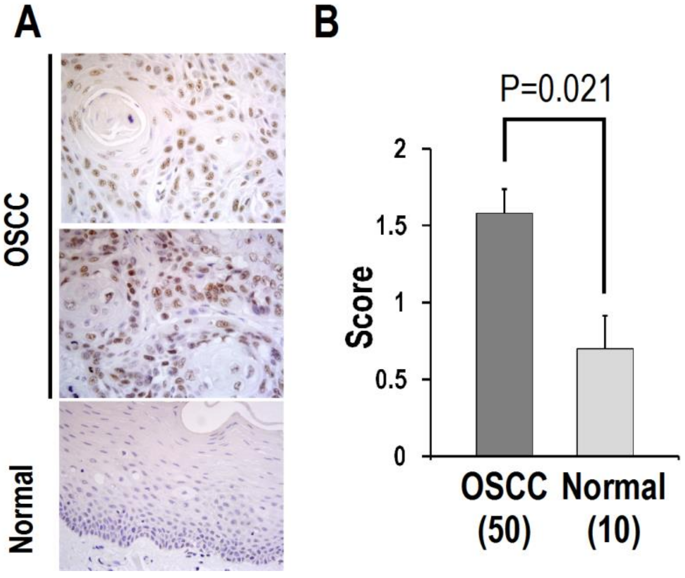

Figure S3

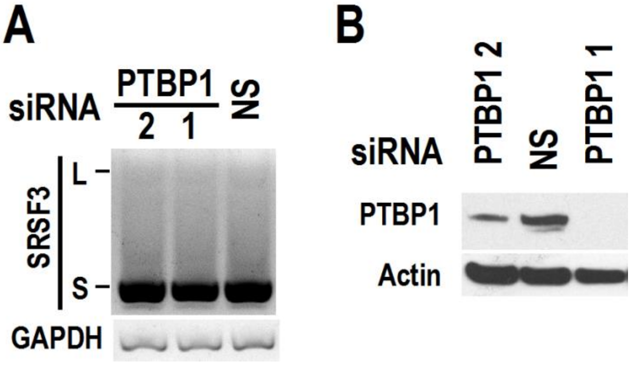

Supplement: Supplementary Figures [file srep14548-s1.pdf]
